# Patient organ doses from megavoltage computed tomography delivery with a helical tomotherapy unit using a general treatment planning system

**DOI:** 10.1093/jrr/rrz005

**Published:** 2019-03-31

**Authors:** Hironori Nagata, Satoru Sugimoto, Hideyuki Hongo, Harumitsu Hashimoto, Yuki Sato, Toru Kawabata, Hiroyuki Watanabe, Tatsuya Inoue, Keisuke Usui, Chie Kurokawa, Keisuke Sasai

**Affiliations:** 1 Department of Radiation Oncology, Graduate School of Medicine, Juntendo University, Bunkyo-ku, Tokyo, Japan; 2 Department of Radiation Oncology, Shonan Kamakura General Hospital, Kamakura, Kanagawa, Japan; 3 Department of Radiology, Shonan Fujisawa Tokusyukai Hospital, Fujisawa, Kanagawa, Japan; 4 Technical Support Group, Radiation Therapy Technical Support Department, Hitachi, Ltd Healthcare Business Unit, Taito-ku, Tokyo, Japan; 5 Department of Radiology, Juntendo University Urayasu Hospital, Urayasu, Chiba, Japan

**Keywords:** helical tomotherapy, MVCT, general treatment planning system, patient-specific organ dose

## Abstract

The purpose of this study was to quantify actual patient organ doses from megavoltage computed tomography (MVCT) using an MVCT beam model of a helical tomotherapy unit in a general treatment planning system (TPS). Dosimetric parameters (percentage depth dose, lateral beam profile, and longitudinal beam profile) of the MVCT beam were measured using Gafchromic EBT3 films (ISP Corporation, Wayne, NJ, USA) and used for beam modeling in a Pinnacle^3^ TPS (Philips, Amsterdam, Netherlands); this TPS is widely used with linear accelerators. The created beam model was adjusted and validated by assessing point doses in a cylindrical phantom in static and helical beam plans with fine, normal and coarse pitches. Maximum doses delivered to important organs from MVCT delivery for five clinical cases were calculated using the created beam model. The difference (average ± one standard deviation for all evaluation points) between calculated and measured doses was −0.69 ± 1.20% in the static beam plan. In the helical beam plan, the differences were 1.83 ± 2.65%, 1.35 ± 5.94% and −0.66 ± 8.48% for fine, normal and coarse pitches, respectively. The average maximum additional dose to important organs from MVCT in clinical cases was 0.82% of the prescribed dose. In conclusion, we investigated a method for quantifying patient organ dose from MVCT delivery on helical tomotherapy using an MVCT beam model in a general TPS. This technique enables estimation of the patient-specific organ dose from MVCT delivery, without the need for additional equipment.

## INTRODUCTION

Helical tomotherapy, a type of intensity-modulated radiation therapy (IMRT), delivers an optimized multidirectional radiation dose to the region of interest using a binary multileaf collimator (MLC) and helical irradiation with the continuous movement of a gantry and couch [1–3]. Accurate positioning of patients is crucial for preventing damage to other organs and tissue in close proximity, due to the steep dose gradient of IMRT. Thus, before treatment delivery, image-guided radiation therapy (IGRT) images from the on-board imaging device are acquired to determine optimal patient placement [4]. For tomotherapy, IGRT using megavoltage computed tomography (MVCT) is performed before each treatment fraction for online set-up correction. However, frequent use of MVCT increases the imaging dose that the patient receives.

Multiple scan average dose (MSAD) is generally used to evaluate MVCT dose during tomotherapy. Typical MSAD values are in the range of 0.6–3.0 cGy, using a water-equivalent phantom [3, 5–7]. However, it is difficult to estimate the actual patient organ dose from the MSAD because it is the point dose at a single representative point in a standard phantom. To quantify organ dose, taking into account the anatomy specific to each patient, a 3D MVCT dose distribution is desirable.

Shah *et al.* reported the dose distribution in a patient from MVCT imaging with a tomotherapy unit [6]. In their study, an MVCT beam model was created using software developed by the manufacturer’s physics group to calculate the 3D dose distribution. The average organ doses of hips, bladder and rectum from MVCT per fraction for prostate cancer treatment were reported to be 1.02, 1.05 and 1.04 cGy, respectively; however, this method is not available to general clinical practice for routine quantification of patient-specific radiation dose. Mege *et al.* also evaluated MVCT doses in a clinical case using Gafchromic films and an anthropomorphic phantom [8]. In their study, three different sites [head and neck, thorax, and pelvis (abdomen)] were assessed. However, they evaluated the dose distribution in a slice at each site in the phantom. These were 2D dose distributions only in representative planes in the phantom, which were not organ volume doses and did not take into account the anatomy specific to each patient.

Radiation dose quantification allows more accurate assessment of the total radiation dose to patients for safer radiotherapy procedures. To quantify the patient-specific dose distribution from MVCT delivery of a tomotherapy unit, we propose a method using a general treatment planning system (TPS). Because in MVCT only a single irradiation field of 400 × 4 mm^2^ is used, the measurement for beam modeling is minimal and can be performed easily at each site. In addition, a nominal energy of 3.5 MV is used in MVCT, which is in the energy region that can be calculated by a general TPS.

In this study, our proposed MVCT beam model of a helical tomotherapy unit was implemented in a commercially available TPS using beam profiles obtained with Gafchromic films. The MVCT beam model was verified with point dose measurements in a phantom. Organ dose data from MVCT in the treatments for typical sites (brain, head and neck, lung, abdomen [pancreas], and prostate) were also estimated.

## MATERIALS AND METHODS

### MVCT on the helical tomotherapy unit

MVCT image acquisition from a helical tomotherapy unit, with the continuous movement of the gantry and couch, is similar to the image acquisition in a helical CT scan. The nominal energy of the incident electron beam is 3.5 MeV. The field size is 400 × 4 mm^2^. The field length (IEC-Y) is 4 mm at the isocenter, and all MLC leaves are open during MVCT delivery. The gantry period per rotation is 10 s. Scan pitch can be selected from three pitch options: fine, normal and coarse. The couch travels per rotation are 4 mm/rotation (fine), 8 mm/rotation (normal) and 12 mm/rotation (coarse), respectively. The user can set the scan pitch and scan range when acquiring MVCT images on the helical tomotherapy unit.

### MVCT beam modeling

**Fig. 1.**
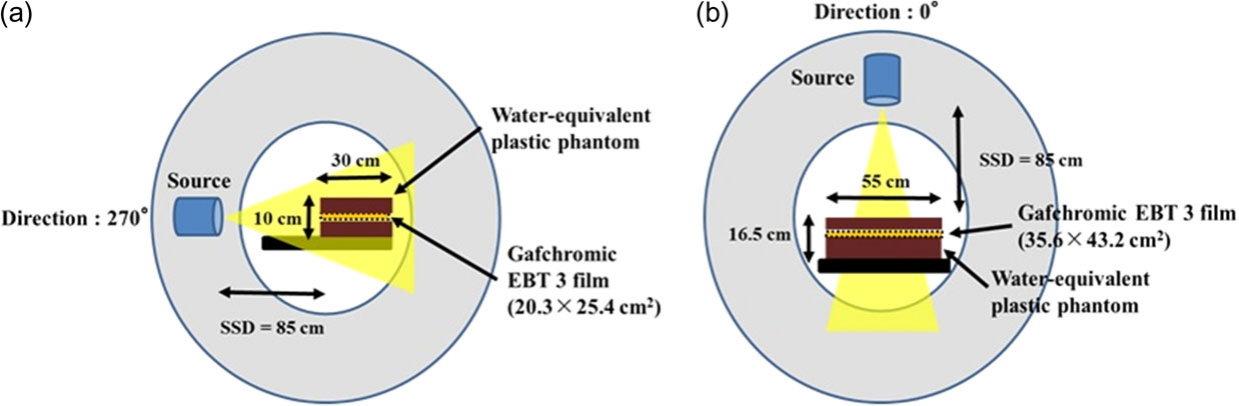
Geometry for the measurement of (a) PDD, (b) OCR_*x*_ and OCR_*y*_. Gafchromic EBT3 films (20.3 × 25.4 cm^2^ for PDD, 35.6 × 43.2 cm^2^ for OCR_*x*_ and OCR_*y*_), and rectangular water-equivalent phantoms were used for the measurements. The films were set at the center for PDD and at a 1.5-cm depth for OCR_*x*_ and OCR_*y*_ in the phantoms.

MVCT imaging percentage depth dose (PDD), lateral beam profile off-center ratio in the *x*-direction (OCR_*x*_), and longitudinal beam profile off-center ratio in the *y*-direction (OCR_*y*_) were acquired to generate the MVCT beam model. Dosimetric parameters were obtained by film measurement using Gafchromic EBT 3 film (ISP Corporation, Wayne, NJ, USA). The geometries for the PDD, OCR_*x*_ and OCR_*y*_ measurements are shown in Fig. 1. PDD, OCR_*x*_ and OCR_*y*_ were measured at a source-to-surface distance (SSD) of 85 cm using rectangular water-equivalent plastic phantoms, which are widely used in tomotherapy units. For the measurement of PDD, the gantry angle was set to 270° and the film (20.3 × 25.4 cm^2^) set at the center of the phantom (width 30 × length 55 × height 10 cm^3^) was irradiated from the side to obtain data in a stable condition. For the profile measurements, the gantry was set to 0° and the film (35.6 × 43.2 cm^2^) set at a 1.5-cm depth of the phantom (width 55 × length 15 × height 16.5 cm^3^) was irradiated from the upper side. The irradiated films were digitized at 75 × 75 dpi^2^ using a film digitizer (Vidar Systems Corporation, Herndon, VA, USA). PDD, OCR_*x*_ and OCR_*y*_ were obtained from the digitized film data using Film Analyzer software (ver. 1.1.2.6; Vidar Systems Corporation). The obtained profiles were exported as csv files and imported into a general TPS for beam modeling. The Pinnacle^3^ TPS (ver. 9.10; Philips, Amsterdam, The Netherlands), which is widely used for modeling of radiotherapy treatment units (not specific to the helical tomotherapy unit), was used in this study. The beam model parameters in Pinnacle^3^, the effective source size, the fluence filter profile, and the energy spectrum were adjusted manually so that the calculated PDD, OCR_*x*_ and OCR_*y*_ reproduced the measured PDD, OCR_*x*_ and OCR_*y*_ in the same manner as the modeling for a conventional linear accelerator (LINAC). A single-field size of 400 × 4 mm^2^, which is used in MVCT, was considered in the beam modeling.

### 3D dose calculation for the general treatment planning system

The 3D dose distribution was calculated to quantify the organ dose in TPS. The helical MVCT irradiation was approximated as the sum of multiple discrete 400 × 4 mm^2^ beams placed at equally separated points as the isocenters with the source-to-axis distance (SAD) of 85 cm. The multiple isocenters were arranged in the longitudinal direction and defined as points of interest (POIs) in the TPS. The beam directions were arranged at 45° intervals (eight beams per rotation). The interval of the POIs was determined from the pitch. The 3D dose distribution is expressed by the following equation:

$$D\left(\vec{x}\right)=\overset{n}{\underset{i=1}{\sum }}{D_{i}}^{400\times 4}\left(\vec{x},{\vec{a}}_{i},g_{i},\text{M}\text{U}\right),$$

where i is the label of the isocenter from 1 to *n* and *n* is the number of the isocenters and varies depending on the pitch and scan length. For example, *n* is 48 (eight beams per rotation × six rotations) in the case of normal pitch (8 mm/rotation) and a 48 mm scan length. ${D_{i}}^{400\times 4}\left(\vec{x},{\vec{a}}_{i},g_{i},MU\right)$ is the dose at a 3D point $\vec{x}$ in a 400 × 4 mm^2^ MVCT beam with the isocenter position ${\vec{a}}_{i}$ and the gantry angle $g_{i}$. MU is a monitor unit (MU) per beam. In the tomotherapy unit, the dose is prescribed using irradiation time, whereas in TPS, the dose is prescribed using MU. To convert irradiation time in MVCT to MU in the TPS, an irradiation time in MVCT of 1 min (60 s) was arbitrarily assigned to 40 MU in the TPS. If the irradiation time in MVCT is *T* s (*T*/60 min), the MU per beam assigned in the TPS becomes 40 × (*T*/60) × (1/*n*). The irradiation time for MVCT can be calculated as (scan length)/(pitch) × (period per rotation). Thus, for example, the prescription MU per beam is 40 × [(48/8 × 10)/60] × 1/48 MU in the case of a normal pitch (8 mm/rotation) and a 48-mm scan length. ${D_{i}}^{400\times 4}\left(\vec{x},{\vec{a}}_{i},g_{i},MU\right)$ is calculated in the TPS using the proposed beam model. The dose calculation algorithm was Convolution/Superposition. To relate the absolute dose delivered in MVCT to MU in the TPS, we performed a calibration procedure for the beam output.

### Beam output calibration

**Fig. 2.**
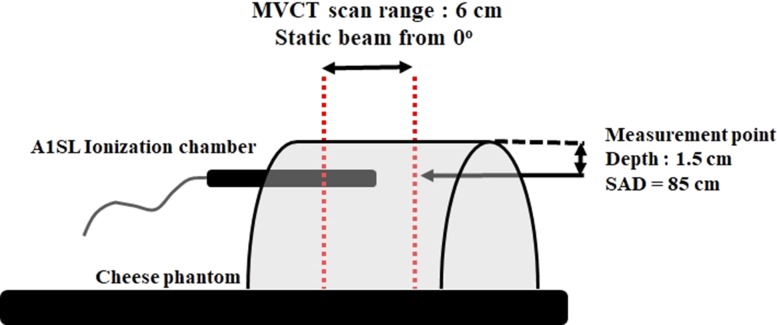
Geometry for the beam output calibration measurement. The point dose was measured at a 1.5-cm depth with a 6-cm scan range irradiated from 0° in the divided cheese phantom.

To relate the MU in the TPS to the MVCT delivery dose, the dose at a 1.5-cm depth in a cylindrical water-equivalent phantom (cheese phantom) was measured using an A1SL ionization chamber (Standard Imaging, Madison, WI, USA). The diameter of the phantom was 30 cm. The phantom was divided in half due to the geometric limitation of the tomotherapy unit, as shown in Fig. 2. A region 6 cm in length was irradiated at a couch speed of 0.1 cm/s, which is the user-settable minimum value, from the 0° direction at a source-to-axis distance (SAD) of 85 cm. The irradiation time was 1 min, corresponding to 40 MU. The A1SL ionization chamber has a collector length of 4.4 mm. Thus, the irradiation range of 6 cm is sufficient to cover the collecting volume. The AAPM TG-51 [9] and TG-148 [3] formalisms were used to measure the absolute point dose. The beam quality conversion factor for point dose measurement was 1.001, which was obtained for the 6 MV treatment beam of the tomotherapy unit using the TG-148 formalism.

**Fig. 3.**
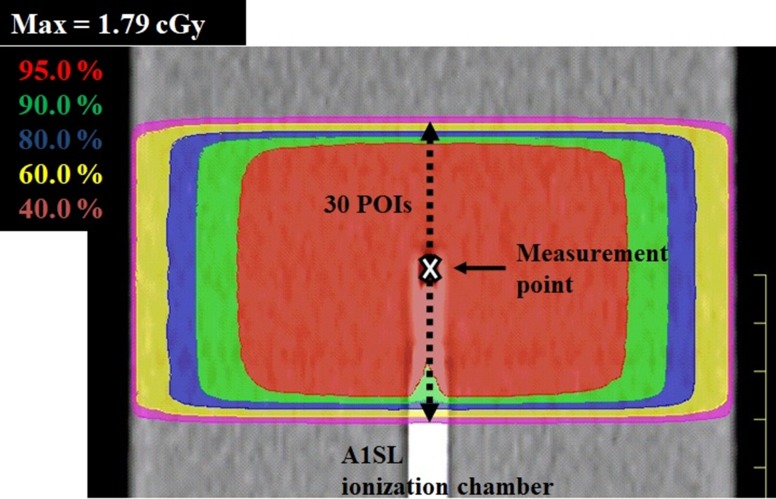
Coronal view of the calculated dose distribution using modeled beam data in the treatment planning system (TPS) for beam output calibration. The 3D dose distribution is calculated as the sum of the dose distribution of 30 beams. The source-to-axis distance (SAD) was 85 cm at a 1.5-cm depth. Isodose lines of the relative value with respect to the maximum point dose are shown. The measurement point is the center of the cavity of the ionization chamber (A1SL) at a 1.5-cm depth. The dose at the measurement point in TPS was compared with the measured dose by A1SL for the determination of the output calibration factor (Dose/MU)_Ref_.

In the TPS, 30 POIs were placed at 0.2-cm intervals (length: 6 cm) in the longitudinal direction on the CT image of the cheese phantom. Beams of 400 × 4 mm^2^ from the 0° direction at SAD 85 cm were placed at the 30 POIs as the isocenters. A 2-s irradiation time was assigned to each beam, corresponding to 40 × (2/60) MU. The 3D dose distribution was calculated as the sum of the dose distribution of the 30 beams (Fig. 3). The output calibration factor in TPS [Dose/MU at the reference condition, (Dose/MU)_Ref_] was adjusted to reproduce the measured dose.

At first, the output calibration factor, (Dose/MU)_Ref_, which is a coefficient to relate the absolute dose to MU in the TPS and corresponds to the dose for 1 MU in the reference condition, was set at 1 cGy/MU. With this setting, the dose at the measurement point was calculated in TPS. The ratio of the measured dose to the calculated dose (*D*_cal,1__cGy/1MU_) was registered in the TPS as (Dose/MU)_Ref._:

$${\left(\text{D}\text{o}\text{s}\text{e}/\text{M}\text{U}\right)}_{\mathrm{Ref}}=\frac{D_{\text{m}\text{e}\text{a}\text{s}}}{D_{\text{c}\text{a}\text{l},1cGy/1\text{M}\text{U}}}$$

With this (Dose/MU)_Ref_, the calculated dose in the reference condition becomes equal to the measured dose.

### MVCT beam model adjustment

**Fig. 4.**
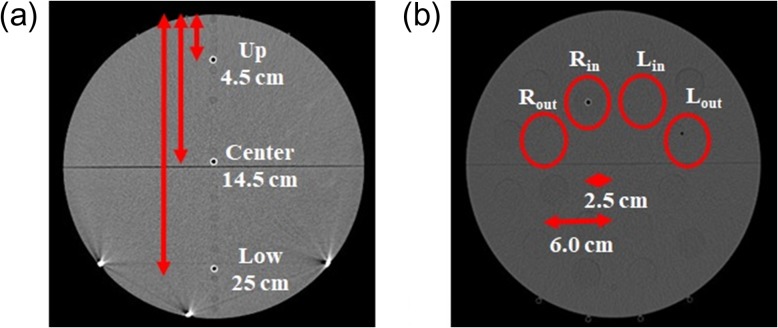
Dose measurement points for MVCT beam model adjustment and validation. (a) Measurement points for static and helical beam plans. The doses at the three central points (up, center and low) were measured. The distances from the surface were 4.5 cm for up, 14.5 cm for center, and 25 cm for low. (b) Measurement points in the static beam plan. The doses of the lateral four points (R_out_, R_in_, L_in_ and L_out_) were measured. The distances from the center were 2.5 cm for R_in_ and L_in_, and 6.0 cm for R_out_ and L_out_. The center of the chamber at each measurement point was set at 3.5 cm outside of the center of the phantom in the longitudinal direction.

**Fig. 5.**
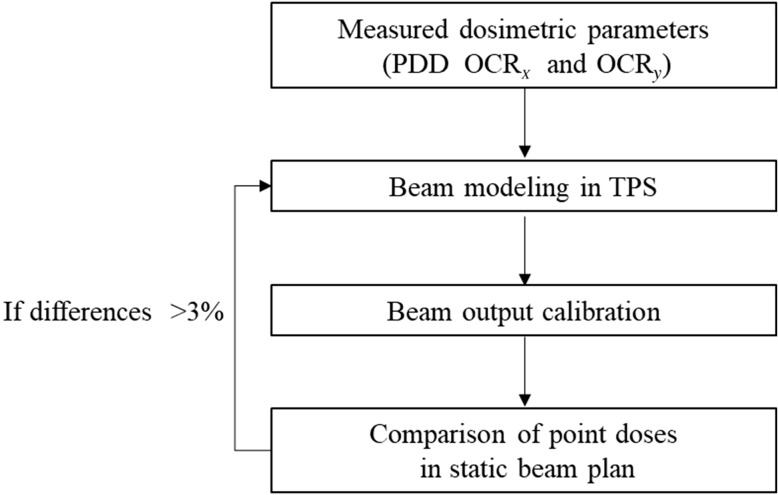
Flow chart for the determination of the megavoltage computed tomography (MVCT) beam model. If the dose difference between measurement and calculation results >3%, the beam model in TPS is modified to make the differences <3%.

The obtained MVCT beam model was adjusted using point dose measurements in the cheese phantom in a static beam plan. An A1SL ionization chamber was used for the measurement. In the static beam plan, the gantry is stationary while the couch is moving. A region, 6 cm in length, was irradiated centering on the chambers at the SAD of 85 cm, with the gantry axis at the center of the phantom. The doses at three central points and four lateral points shown in Fig. 4a and b were measured and compared with the calculated doses. The modeled MVCT beam was adjusted by tuning the energy spectrum so that the differences between measurement and calculation results were within 3% for all points. The flow chart for determination of the MVCT beam model is shown in Fig. 5.

### Validation of the MVCT beam model

The adjusted MVCT beam model was validated by assessing point doses in the cheese phantom using an A1SL ionization chamber in helical beam plans. The helical beam plans included a 4.8-cm irradiation range with moving gantry and couch set-up for helical exposure. Point doses were evaluated with the pitches of fine, normal and coarse. The irradiation times for point dose measurement were 120, 60 and 40 s for fine, normal and coarse pitches, corresponding to 80 MU, 40 MU and 26.7 MU, respectively. In the calculation, multiple isocenter POIs were set on the axis of rotation of the tomotherapy unit, and the beam directions were arranged at 45° intervals (eight beams per rotation). The intervals of the POIs were set to 0.05, 0.1 and 0.15 cm for fine, normal and coarse pitches, respectively. Three central point doses, shown in Fig. 4a, were measured and compared with the calculated doses.

### MVCT dose estimation for clinical cases

**Fig. 6.**
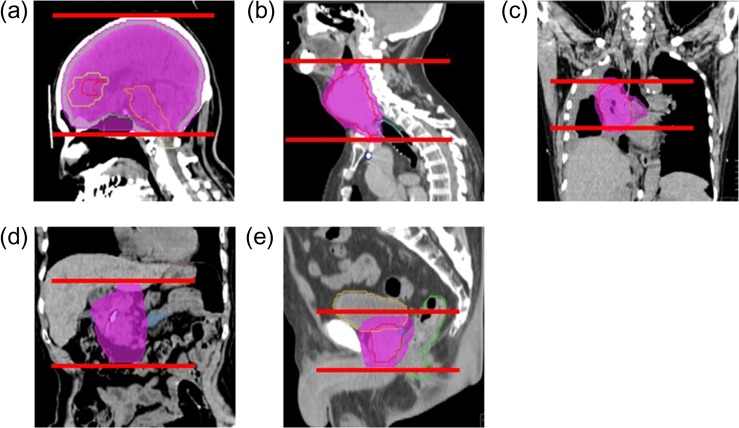
CT images and scan areas of five clinical cases for evaluating the organ dose from MVCT delivery: (a) brain, (b) head and neck, (c) lung, (d) abdomen (pancreas) and (e) prostate. The scan areas are from the superior edge to the inferior edge of the planning target volume (PTV, magenta area).

The absorbed doses to organs of interest from MVCT delivery for five clinical cases: brain, head and neck, lung, abdomen (pancreas), and prostate, were estimated using the created MVCT beam model. Figure 6 shows CT images of the clinical cases and scan ranges for the MVCT used in the MVCT dose calculation, from the superior edge to the inferior edge of the planning target volume (PTV).

Dose estimation was performed for organs in the irradiated range of MVCT [brain; brain stem; right (R) and left (L) eyes; R and L parotids; oral cavity; spinal cord; R and L lungs; heart; stomach; R and L kidneys; duodenum; bladder; both femoral heads (FHs); rectum; and prostate]. All organ doses were calculated with normal pitch. In the MVCT dose calculation, the intervals of isocenter POIs were 0.1 cm, and the beam directions were arranged at 45° intervals (eight beams per rotation). The maximum dose per fraction and the maximum dose for the total fraction of each organ were calculated.

## RESULTS

### MVCT beam modeling and beam output calibration

**Fig. 7.**
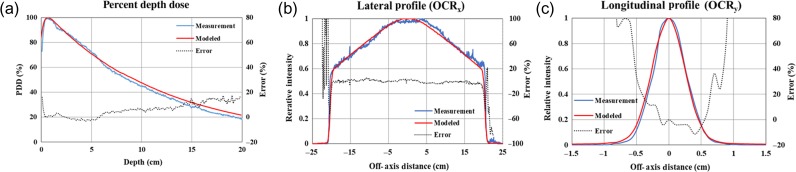
The profiles modeled in TPS (red solid line) and measured using films (blue solid line) for the MVCT 400 × 4 mm^2^ field: (a) percentage depth dose (PDD), (b) lateral beam profile (OCR_*x*_), and (c) longitudinal beam profile (OCR_*y*_). The errors between the model and measurement (black dotted line) are also shown. OCR_*x*_ and OCR_*y*_ were acquired at a 1.5-cm depth.

The doses calculated using the created MVCT beam model and the adjusted beam output calibration factor were compared with the point dose measurement in the static beam plan in Table 1. The differences between the calculated and measured doses were >3% at the central center (−5.34%), the central low (−9.84%), the lateral R_out_ (−4.37%), and the lateral L_out_ (−3.48%). To make the difference <3%, the energy spectrum in the high-energy region (∼ 3 MeV) in the beam model was modified to increase the calculated dose in a deep region, because the dose difference at the central low point in Fig. 4a was particularly large. With the modification, the calculated PDD became 1.92% larger at a depth of 15 cm than that without modification. The comparison of point doses in static beam plans after modification is shown in Table 2. The differences [average ± 1 standard deviation (SD)] between the measurement and the calculation were −1.42 ± 1.35% for the three central points and −0.14 ± 0.86% for the four lateral points. The maximum deviation was <2.5%. The measured and calculated PDD, OCR_*x*_ and OCR_*y*_ after modification are shown in Fig. 7. The beam output calibration factor (Dose/MU)_Ref_ was 0.40 cGy/MU. The modified MVCT beam model was adopted for calculating the 3D dose distribution.

**Table 1.** Dose comparisons between the measurement and calculation in the static beam plan before energy spectrum modification

	Central points				
	TPS (cGy)	Measurement (cGy)	Difference (%)	Average (%)	SD (%)
Up	1.68	1.70	−0.79	−5.32	4.53
Center	0.92	0.98	−5.34		
Low	0.44	0.49	−9.84		
	Lateral points				
	TPS (cGy)	Measurement (cGy)	Difference (%)	Average (%)	SD (%)
R_in_	1.31	1.33	−1.19	−2.73	1.45
R_out_	0.99	1.04	−4.37		
L_in_	1.32	1.34	−1.89		
L_out_	1.00	1.04	−3.48		

TPS = treatment planning system, SD = standard deviation.

**Table 2.** Dose comparisons between the measurement and calculation in the static beam plan after energy spectrum modification

	Central points				
	TPS (cGy)	Measurement (cGy)	Difference (%)	Average (%)	SD (%)
Up	1.70	1.70	0.10	−1.42	1.35
Center	0.96	0.98	−1.85		
Low	0.48	0.49	−2.50		
	Lateral points				
	TPS (cGy)	Measurement (cGy)	Difference (%)	Average (%)	SD (%)
R_in_	1.34	1.33	0.85	−0.14	0.86
R_out_	1.02	1.04	−1.18		
L_in_	1.35	1.34	0.20		
L_out_	1.04	1.04	−0.41		

TPS = treatment planning system, SD = standard deviation.

### Validation of MVCT beam model

In the helical beam plans for validating the beam model, the measured and calculated doses at the three central points were compared for three pitches (fine, normal and coarse). The differences are shown in Table 3. The average differences and standard deviations were 1.83 ± 2.65% for fine pitch, 1.35 ± 5.94% for normal pitch and −0.66 ± 8.48% for coarse pitch. As the pitch increased, a large deviation was observed. The maximum absolute difference was 0.08 cGy for the fine pitch, 0.08 cGy for the normal pitch and 0.06 cGy for the coarse pitch.

**Table 3.** Dose comparison between the measurement and calculation in the TPS with the helical beam plans for validating the MVCT beam model

	Fine				
	TPS (cGy)	Measurement (cGy)	Difference (%)	Average (%)	SD (%)
**Up**	**2.32**	**2.35**	−**1.23**	**1.83**	**2.65**
**Center**	**2.22**	**2.14**	**3.48**		
**Low**	**2.33**	**2.26**	**3.24**		
	**Normal**				
	**TPS (cGy)**	**Measurement (cGy)**	**Difference (%)**	**Average (%)**	**SD (%)**
**Up**	**1.31**	**1.23**	**6.29**	**1.35**	**5.94**
**Center**	**1.12**	**1.09**	**2.99**		
**Low**	**1.02**	**1.08**	−**5.24**		
	**Coarse**				
	**TPS (cGy)**	**Measurement (cGy)**	**Difference (%)**	**Average (%)**	**SD (%)**
**Up**	**0.99**	**0.93**	**6.88**	−**0.66**	**8.48**
**Center**	**0.75**	**0.74**	**0.97**		
**Low**	**0.56**	**0.62**	−**9.83**		

TPS = treatment planning system, MVCT = megavoltage computed tomography, SD = standard deviation.

### MVCT dose estimation for clinical cases

**Fig. 8.**
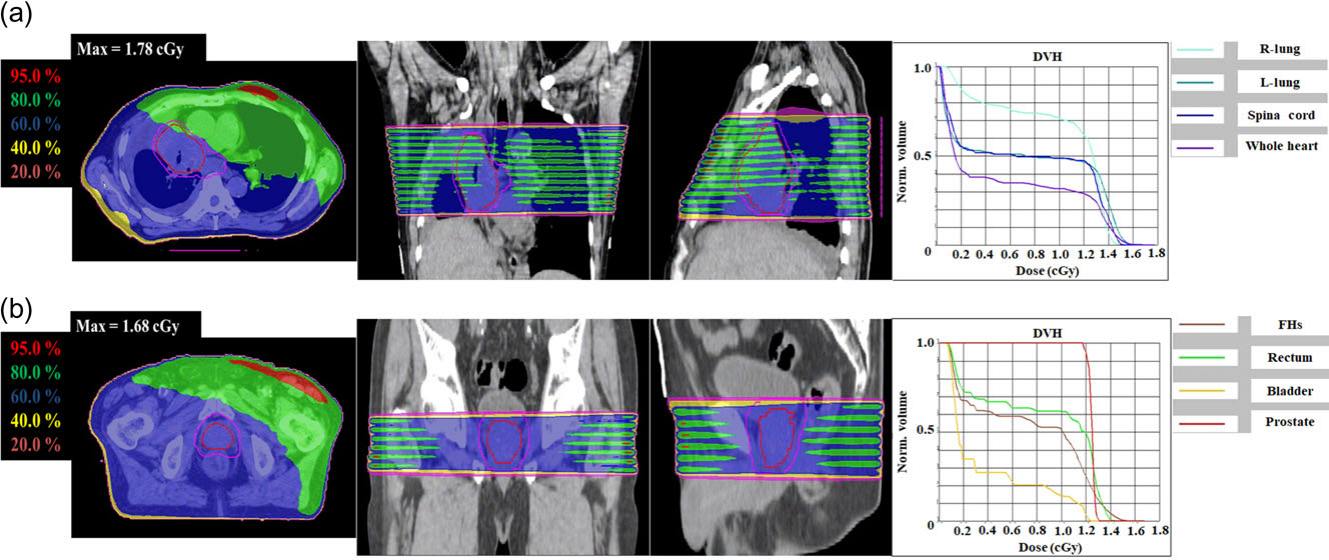
Dose distributions (transverse, coronal and sagittal) and dose–volume histograms (DVHs) for (a) a lung case and (b) a prostate case. Relative doses are normalized to the maximum dose as 100%.

The maximum absorbed doses delivered to organs of clinical interest from MVCT delivery were calculated using the created MVCT beam model in the TPS. One-fraction and total maximum absorbed doses of each organ from the MVCT delivery with normal pitch are shown in Table 4 with the prescription doses. For the brain case, the maximum additional dose to each organ from MVCT was ~0.6% of the prescribed dose. For the head and neck case, the maximum additional dose to the R parotid was largest among all organs evaluated and 1.06% of the prescribed dose. The total maximum additional doses were high because the neck region is anatomically thin and the number of fractions is large for the head and neck case. In the lung, abdomen and prostate cases, the organs with the highest total maximum additional dose were L lung, stomach, and FHs, with 55.34 cGy, 47.60 cGy and 58.22 cGy, respectively. The average maximum additional dose over all organs of interest was 0.82% of the prescribed dose. The 3D dose distribution and dose–volume histogram (DVH) in the lung and prostate cases, which were used to obtain the maximum absorbed doses, are shown in Fig. 8 as examples. High-dose areas appeared near the surface of the body in the calculated 3D MVCT dose distribution.

**Table 4.** Maximum absorbed doses of organs of interest from MVCT delivery with normal pitch

Region	Organ of interest	Prescription^a^	Max dose/fx (cGy)^b^	Total Max dose (cGy)^c^	Percentage of additional dose (%)^d^
Brain		300 × 10			
	Brain		1.88	18.80	0.63
	Brain stem		1.66	16.57	0.55
	R-eye		1.77	17.67	0.59
	L-eye		1.84	18.40	0.61
Head and neck		180 × 39			
	L-parotid		1.62	63.14	0.90
	R-parotid		1.92	74.72	1.06
	Oral cavity		1.86	72.35	1.03
	Spinal cord		1.73	67.51	0.96
Lung		180 × 33			
	L-lung		1.68	55.34	0.93
	R-lung		1.55	51.18	0.86
	Spinal cord		1.51	49.96	0.84
	Heart		1.63	53.79	0.91
Abdomen		180 × 28			
	Stomach		1.70	47.60	0.94
	R-kidney		1.65	46.20	0.92
	L-kidney		1.59	44.38	0.88
	Spinal cord		1.53	42.92	0.85
	Duodenum		1.60	44.88	0.89
Prostate		200 × 38			
	Bladder		1.24	47.27	0.62
	FHs		1.53	58.22	0.77
	Rectum		1.44	54.68	0.72
	Prostate		1.31	49.82	0.66

MVCT = megavoltage computed tomography, FHs = femur heads. ^a^The prescribed dose (prescription) is shown as (fraction dose [cGy]) × (number of fractions). ^b^Max dose/fx is the maximum dose per fraction to each organ of interest. ^c^Total Max dose is Max dose/fx multiplied by the number of fractions. ^d^Percentage of additional dose is Total Max dose divided by the total prescribed dose.

## DISCUSSION

In the tomotherapy unit, IGRT using MVCT for each fraction is important for accurate dose delivery to a target. However, because frequent use of MVCT increases the additional dose to normal organs, the absorbed dose to these organs from MVCT delivery should be estimated. For patient safety, knowing the IGRT dose is highly important. Therefore, in this study, a method for quantifying actual patient organ dose using an MVCT beam model of a tomotherapy unit in a general TPS was developed and verified.

Dosimetric data were acquired by film measurement for MVCT beam modeling and imported into Pinnacle^3^, a widely used TPS. In a previous study, Shah *et al.* used a 2D diode array [232 diodes; detector spacing, 5 mm (4 mm) along the lateral (longitudinal) profile direction] to acquire dosimetric data of MVCT on the tomotherapy unit [6]. The measurement with such a diode array is simple; however, the spacing between diodes is relatively large. Therefore, the resolutions of the acquired PDD and OCR data using the diode array are limited.

In contrast, film measurements offer high resolution. Moreover, the data acquisition is simple and efficient compared with measurement in a water tank using an ionization chamber. Chen *et al.* conducted film measurements using Kodak X-Omat V film to acquire an MVCT beam profile of the tomotherapy unit. They reported that the difference between the measurement and calculation was ~0.05 cGy, and the thread effect was well matched [10]. Therefore, film measurement is suitable for obtaining MVCT beam data on the tomotherapy unit.

In the tomotherapy system, the modeling of the MVCT beam cannot be performed in the TPS on the user side; however, the MVCT beam can be modeled easily in a general TPS using our method. The necessary measurements for MVCT beam modeling are the two film irradiations for PDD and the OCRs, one chamber measurement for beam output calibration, and several chamber measurements for validation of the created beam model. After beam modeling, the IGRT dose can be obtained in the same manner as that used for dose calculation in a normal treatment plan. The method proposed in this study can easily quantify patient organ doses from MVCT delivery at each site. In addition, this method can be applied to other MVCT radiation therapy units with a general TPS.

The created beam model was adjusted and verified with static and helical beam plans by point dose assessment. In this study, for the dose measurement with a 3.5-MV MVCT beam, the beam quality conversion factor was taken as the same value as the 6 MV treatment beam. The reason for this is that Jeraj *et al.* reported that the average energy of the MVCT beam is close to that of treatment beam [11]. In a study by Mege *et al.*, the conversion factor for measuring the point dose from MVCT delivery was taken as that for the 6 MV treatment beam due to the small difference between the average energy of the MVCT beam and the treatment beam [8]. In AAPM TG-148, no correction of the conversion factor for the treatment beam to use for the imaging beam was recommended [3]. Therefore, the conversion factor for the treatment beam was adopted as that for the MVCT beam in this study.

In the static beam plan for beam model adjustment, a scan length of 6 cm and a couch speed of 0.1 cm/s were adopted in this study. To investigate the influence of the scan length on the beam modeling, the scan length was changed from 6 cm to 12 cm and 3 cm with a couch speed of 0.1 cm/s. The measured point doses increased (12 cm) and decreased (3 cm) by ~2.7% with respect to a 6-cm scan length due to scattered radiation. The calculated point doses in TPS also increased (12 cm) and decreased (3 cm) by ~2.1% with respect to a 6-cm scan length. These results indicate that the change in scatter radiation due to the change in scan length can be considered in the TPS. The uncertainty from the change in scan length was estimated as <1%. The couch speed does not affect the beam modeling because the change in couch speed only causes a change in irradiation time, which can be considered exactly by changing the MU proportionally in TPS. When the couch speed was varied to 0.1 cm/s (40 MU), 0.2 cm/s (20 MU) and 0.3 cm/s (13.3 MU) with a scan length of 6 cm, both the measured point doses and the calculated point doses in the TPS decreased by 50% for 0.2 cm/s and 67% for 0.3 cm/s with respect to a 0.1-cm/s couch speed.

In the helical beam plan for validating the created beam model, a scan length of 4.8 cm was applied in this study. To investigate the influence of the change in scan length, the scan length was changed from 4.8 cm to 9.6 cm and 2.4 cm with normal pitch. The measured point doses at the center increased by 15.8% for 9.6 cm and decreased by 8.3% for 2.4 cm, compared with the 4.8 cm scan length due to scattered radiation. The calculated point doses at the center in TPS increased by 12% for 9.6 cm and decreased by 11% for 2.4 cm, with respect to the 4.8 cm scan length. There were ~3% differences between the measurements and calculations in TPS. However, the absolute difference was ~0.04 cGy, which was not a significant difference. These results indicate that the change in scatter radiation due to the change in scan length can be considered in TPS in helical beam plans. Thus, the conditions (scan length and couch speed) for the static and helical beam plans in this study do not significantly affect the results because the variation in the doses with changes in the measurement conditions are reproduced in the TPS.

For the helical beam plan, the relatively large gantry interval (eight beams per rotation) was adopted in the TPS. When the gantry interval changed to 16 beams per rotation, the change in the calculation point doses was ~0.65%, which was not significant. In addition, when pitch changes, the dose divergence in the *y*-direction changes with helical irradiation due to the change in couch speed [Tomotherapy Incorporated: MVCT Imaging with J1 TomoImage Beam, Application Note 105925 Rev. A. 2010 (unpublished)]. In our proposed method, helical irradiation was divided into beams from multiple directions with different beam centers to simulate helical irradiation. The effect of changing the couch speed is partially included by changing the interval between the beam centers. Actually, the rate of reduction in the point doses at the center when the pitch changed from fine to normal and coarse was ~50% for normal and ~66% for coarse in both the measurement and calculation, as shown in Table 3. This indicates that the effect of changing the dose divergence in the *y*-direction on dose distribution is taken into account in our method.

In adjusting the beam model, the energy spectrum was modified so that the differences between the measured and calculated doses in the static beam plan became <3% at all dose-evaluated points. After the energy spectrum modification, there was a deviation between the measured and calculated PDDs in a deep region. This can be explained by a difference in the phantom sizes between the film measurement and modeling in the TPS, the depth-scaling factor of the rectangular water-equivalent plastic phantom, the inherent uncertainty of the film measurement, and the inherent uncertainty of modeling for a narrow beam [6].

In the static beam plan, the differences between measurement and calculation were within 2.5% at all points and showed good agreement after energy spectrum modification. In the helical beam plans, as the pitch increased, a large deviation of 8.5% (1 SD) was observed. In a previous study, the difference between measurement and calculation results for a helical beam plan was within 4% and smaller than that of this study [6]. One reason for this is that, in the dose calculation method in this study, helical irradiation was divided into relatively limited beams of conventional irradiation (separated isocenters in the longitudinal direction and eight beams per rotation). The other reason is that the influence of the couch was not considered in this study. However, the absolute systematic errors were small, of the order of 0.05 cGy. Therefore, the proposed MVCT beam model offers clinically acceptable accuracy for IGRT dose management.

The average total maximum additional dose to important organs from MVCT delivery in clinical cases was 47.40 cGy and 0.82% of the prescribed dose for normal pitch. The maximum additional fraction dose of the R parotid in the head and neck region, 1.92 cGy/fx, was the largest of all the organs. Because the neck is a thin structure, the doses absorbed by organs increase with helical irradiation of the same dose rate, pitch, and speed per rotation. In addition, the doses to organs near the surface of the body were high. The additional doses to important organs were estimated for the normal pitch; if delivered with a fine (coarse) pitch, the absorbed organ dose is expected to increase (decrease).

In comparison with a previous study by Shah *et al.* [6], the maximum additional doses in this study were ~10% higher in all organs. One reason for this difference is the difference in the MVCT dose level measured. The measured center point dose with the normal pitch in the helical beam in the cheese phantom in Table 3 was 2.8% higher than that reported by Shah *et al.* There is uncertainty in the comparison with the MVCT dose level reported by them, because the scan length is not mentioned in their article. The scan length for the measurement of the MVCT dose level recommended in the TG-148 [3] is a length that completely covers the phantom. In the case of the cheese phantom, the scan length was 18 cm. In the study by Mege *et al.* [8], a scan length of 10 cm was adopted for the measurement of the MVCT dose level. We used a relatively short scan length of 4.8 cm. We measured the center doses in the cheese phantom with scan lengths of 10 cm and 18 cm and compared them with the result of Shah *et al.* The measured center doses for 10-cm and 18-cm scan lengths were 16% and 22.2% higher than those reported by them, respectively. Thus, our MVCT dose level was expected to be 2.8–22.2% higher than their dose levels. The difference in the MVCT dose level explains the organ dose difference between Shah *et al.*’s results and our results.

Our results (the maximum absorbed doses to parotids, lungs and bladder) were compared with the MSADs reported by Mege *et al.* [8], which were delivered to three clinical sites (head and neck, thorax, and pelvis [abdomen]). Our results were 17.2% lower than the average MSADs reported in Mege *et al.* One reason for this difference is the difference in the MVCT dose levels. The measured center dose for a 10-cm scan length in the cheese phantom was 10.9% lower than that reported by them. The other reason is the difference in the types of phantoms (anthropomorphic phantom vs patient-specific CT image). However, our results were almost within the MSADs range they reported, which supports the validity of our method.

In the lung region, heterogeneity should be considered in MVCT dose calculations. Because the dose calculation algorithm in TPS was Convolution/Superposition, the calculation can be performed with high accuracy, even in heterogeneous regions such as the lungs [12]. The maximum absorbed lung dose with normal pitch in this study was 19% higher than that of Shah *et al.* and 16.2% lower than the average MSAD reported by Mege *et al.* These differences can be explained by the differences in the MVCT dose levels, as mentioned before. The difference in the arm position in the CT images also contributes to the lung dose difference between Shah *et al.* (hands down) and us (hands up). This difference is another reason that the maximum absorbed lung dose in our study was higher than that of Shah *et al.* Our result was within the MSAD range reported by Mege *et al.* This indicates that heterogeneity in the lung region can be considered using our method.

**Fig. 9.**
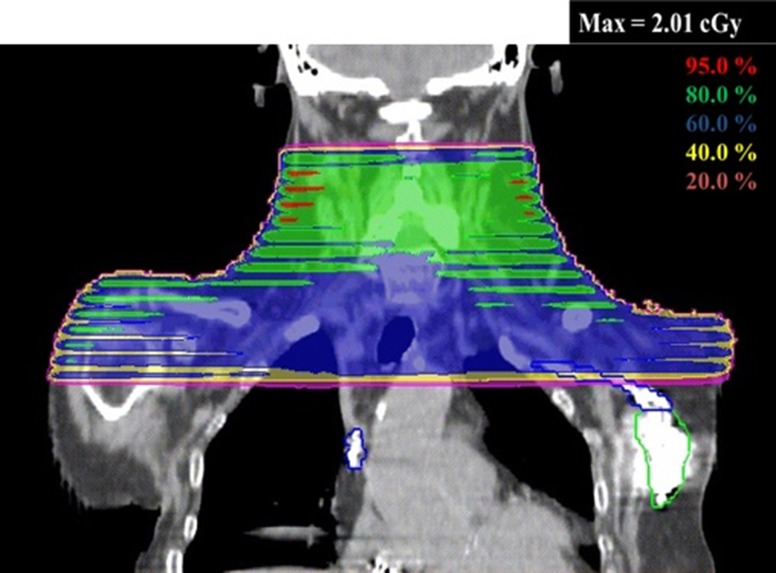
The dose distribution of the coronal view in a head and neck region including a pacemaker (green line contour). It is possible to quantify the MVCT dose to the pacemaker. The maximum additional fraction dose of the pacemaker was 0.08 cGy/fx. Relative doses are normalized to the maximum dose as 100%.

Because 3D dose distribution data can be output as DICOM-RT using this technique, a treatment plan can be evaluated with 3D dose distribution, including IGRT dose, using a treatment planning support system. To evaluate the safety aspects of a treatment plan, the doses to organs for which the maximum dose should be considered, such as the spinal cord, can be obtained, including the IGRT dose from MVCT. Additionally, this technique is useful for determining the delivered MVCT imaging dose and range for patients with a pacemaker or implantable cardioverter defibrillator as shown Fig. 9. It will also be effective in radiation therapy for pediatric patients, whose organ doses have to be particularly carefully considered for secondary cancer risk.

In this study, we developed a method for quantifying patient organ doses from MVCT delivery on a helical tomotherapy unit using an MVCT beam model in a general TPS. Dosimetric MVCT data were acquired by film measurement and modeled in Pinnacle^3^, which is widely used for modeling of treatment units in radiotherapy institutes to calculate 3D dose distribution. The modeled beam data were adjusted and verified by point dose assessments in static and helical beam plans. The accuracy was within 8.5% for 1 SD; the absolute systematic error was ~0.05 cGy. Additional MVCT doses delivered to organs of clinical interest were also calculated using our MVCT beam model. The average maximum additional dose over all organs of interest was 0.82% of the prescribed dose. This technique is valuable for the management of IGRT dose for patient safety, as patient-specific organ doses from MVCT delivery can be estimated at each site.
